# Biocatalytic potential of vanillin aminotransferase from *Capsicum chinense*

**DOI:** 10.1186/1472-6750-14-25

**Published:** 2014-04-09

**Authors:** Nora Weber, Abdelrahman Ismail, Marie Gorwa-Grauslund, Magnus Carlquist

**Affiliations:** 1 Division of Applied Microbiology, Department of Chemistry, Lund University, SE-22100 Lund, Sweden; 2 Centre for Analysis and Synthesis, Department of Chemistry, Lund University, SE-22100 Lund, Sweden

**Keywords:** Putative aminotransferase, PAMT, VAMT, Transaminase, Vanillylamine, 1-phenylethylamine, Acetophenone, Whole-cell biocatalysis, Capsaicinoids

## Abstract

**Background:**

The conversion of vanillin to vanillylamine is a key step in the biosynthetic route towards capsaicinoids in pungent cultivars of *Capsicum* sp. The reaction has previously been annotated to be catalysed by PAMT (putative aminotransferase; [GenBank: AAC78480.1, Swiss-Prot: O82521]), however, the enzyme has previously not been biochemically characterised *in vitro*.

**Results:**

The biochemical activity of the transaminase was confirmed by direct measurement of the reaction with purified recombinant enzyme. The enzyme accepted pyruvate, and oxaloacetate but not 2-oxoglutarate as co-substrate, which is in accordance with other characterised transaminases from the plant kingdom. The enzyme was also able to convert (*S*)-1-phenylethylamine into acetophenone with high stereo-selectivity. Additionally, it was shown to be active at a broad pH range.

**Conclusions:**

We suggest PAMT to be renamed to VAMT (vanillin aminotransferase, abbreviation used in this study) as formation of vanillin from vanillylamine could be demonstrated. Furthermore, due to high stereoselectivity and activity at physiological pH, VAMT is a suitable candidate for biocatalytic transamination in a recombinant whole-cell system.

## Background

Chili pepper fruit (*Capsicum* sp.) gives rise to a strong pungent sensation when eaten, due to the presence of capsaicinoids, with capsaicin (*trans*-8-methyl-*N*-vanillyl-6-nonenamide) as the most known example [1]. Capsaicinoids have been considered as anti-obesity, antimicrobial and antineoplastic agents [1,2], and for the prevention of hypertension [3]. Additionally, capsaicin is a very potent agonist of the pain receptor TRPV1 [4,5] and is therefore used as a therapeutic drug in the treatment of peripheral neuropathy. Since the discovery of capsaicinoids there has been substantial amount of research dedicated to the understanding of biosynthetic pathways leading to capsaicinoids in different cultivars of *Capsicum* sp. Although the biochemical pathways leading to capsaicin are described, there is still a knowledge-gap with regards to the identity of some of the involved enzymes (cf. review by Aza-González *et al*. [2]).

**Figure 1:**
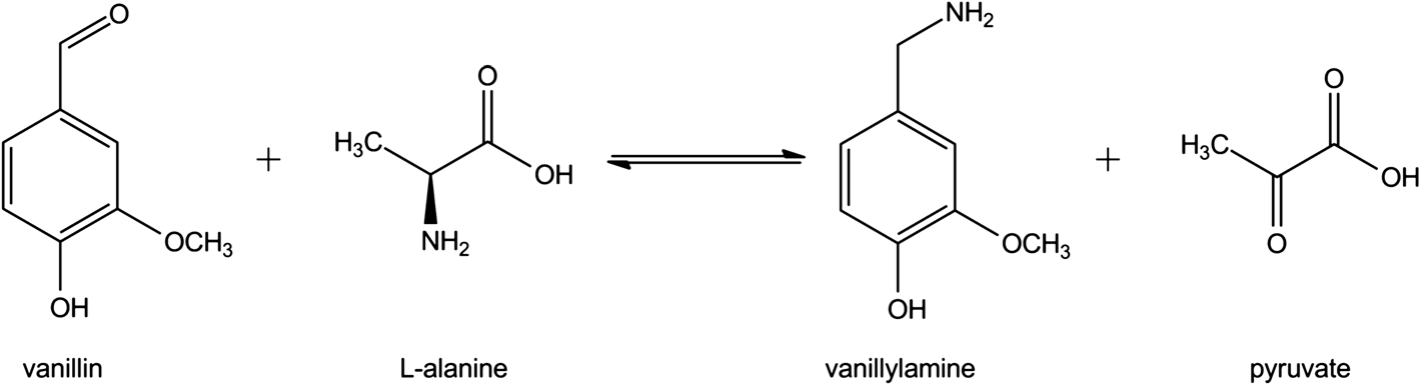
Transamination of vanillin to vanillylamine with L-alanine as amine donor, catalysed by vanillin transaminase (VAMT).

In brief, capsaicin synthesis in *Capsicum* sp. is achieved through combination of two separate pathways, namely: (i) phenylalanine conversion to vanillylamine; and (ii) valine conversion to 8-methyl-6-nonenyl-CoA acid via the fatty acid metabolism. The two pathways are connected in a capsaicinoid synthase-catalysed condensation reaction between vanillylamine and 8-methyl-6-nonenyl-CoA to form capsaicin [2]. A key step in the biosynthetic route to capsaicin is the conversion of vanillin to vanillylamine via transamination of the aldehyde moiety into the corresponding amine [6] (Figure 1). The responsible enzyme for transamination of vanillin has previously been addressed to be putative aminotransferase (PAMT) [GenBank: AAC78480.1, Swiss-Prot: O82521] [7]. Although functional studies *in vivo* clearly indicate that PAMT is responsible for the transamination of vanillin in *Capsicum* sp. [6,8], the pure enzyme has, to the best of our knowledge, not been characterised *in vitro*.

In this study, the gene encoding PAMT was functionally expressed in *Escherichia coli* and the polyhistidine-tagged enzyme was purified to homogeneity using affinity chromatography and subsequently characterised. It is confirmed that PAMT converts vanillylamine to vanillin, and should therefore from now on be called "vanillin aminotransferase (VAMT)". The enzyme can also be used for the kinetic resolution of *racemic* 1-phenylethylamine to (*R*)-1-phenylethylamine and acetophenone, and thus carries the potential to be applied for biocatalytic transamination for the preparation of chiral amines.

## Results and discussion

### Cloning, expression and purification of VAMT

**Figure 2:**
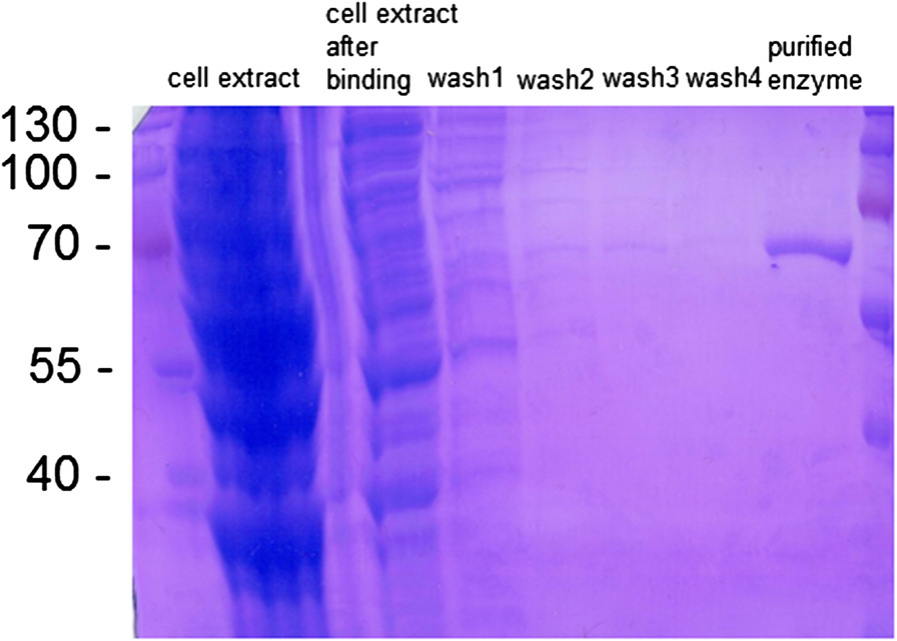
**SDS-PAGE analysis of cell extract, purification steps and purified VAMT.** Molecular size of VAMT with His-tag: 57.5 kDa.

Codon-optimised synthetic *C. chinense* VAMT gene was cloned into an *E. coli* expression vector downstream of the IPTG-inducible T7 promoter, resulting in plasmid pNW1 (Table 1). The plasmid was used to transform *E. coli* BL21, generating strain TMB2100 (Table 1). At first the expression of the protein was investigated with induction at optimal temperature for *E. coli* growth (37°C), however, no enzyme activity in cell free lysate could be registered. Possible explanations include the formation of inclusion bodies or other incorrect folding of the recombinant protein as previously observed for other systems [9]. To solve this problem, a lower temperature (15°C) for the protein induction-expression step was chosen, which led to a high expression of the active enzyme. The polyhistidine-tagged protein was purified from cell free lysate using affinity chromatography, and no contaminating proteins could be detected by SDS-PAGE analysis of the purified enzyme solution (Figure 2).

**Table 1:** Strains and plasmids used in this study

**Strains and plasmids**	**Relevant phenotype**	**Reference**
**Plasmids**		
pUC 57 VAMT	Gene for *VAMT*	GenScript, NJ, USA
pRSETB	Plasmid with T7 promotor and terminator, *Amp* resistance gene	Invitrogen, CA, USA
pNW1	*VAMT* under T7 promoter, with T7 terminator, *Amp* resistance gene	This study
**Strains**		
*E. coli* DH5α		Life Technologies, MD, USA
*E. coli* BL21(DE3) pLys		Invitrogen, CA, USA
*E. coli* TMB2100	*E. coli* BL21, containing pNW1	This study

### Functional validation of vanillin aminotransferase activity

Most known transaminases of both bacterial and plant origin accept L-alanine as amine donor [10-15]. The purified enzyme was therefore first evaluated for the conversion of vanillin to vanillylamine with a high excess of alanine as amine donor. It was, however, not possible to detect any formation of vanillylamine. Conversion was neither observed with the following amine donors: D-alanine, GABA, glutamine and ornithine. However it cannot be excluded that some vanillylamine (below the detection limit of our HPLC analysis) was formed since transamination of a small amount of vanillin was previously observed in crude cell extract from pungent pepper *Capsicum annuum* and with GABA as amine donor [6]. However, the Gibbs free energy of the reaction, ΔG0’, as calculated by using the group contribution method [16], was ΔG0’ = +2.55 kcal/mol when using L-alanine and other amino acids as amine donor. The reaction was consequently thermodynamically favouring the reverse reaction and a high conversion of vanillin was, even with an excess addition of the amine donor, not expectable. It has previously been shown for other transaminases that a (co-) product removal system was required to reach conversion of aromatic ketones to amines [12,17,18]. Therefore, the enzyme was instead evaluated for the conversion of vanillylamine to vanillin using pyruvate as amine acceptor. In this case, the formation of vanillin could be detected shortly after the start of the reaction (the specific activity of the enzyme was approximately 5 U/mg protein), which indicates that VAMT indeed is responsible for vanillylamine formation in *C. chinense*. Whether this activity can be found in orthologous plant transaminases and is a general trait, or if it is a specific natural function of VAMT that has been evolved over time, is unknown. BlastP search within the NCBI database ( http://blast.ncbi.nlm.nih.gov/Blast.cgi) with the amino acid sequence of VAMT identified γ-aminobutyrate (GABA) transaminases, catalysing the conversion of GABA to succinate semialdehyde as the most similar enzymes. The amino acid sequence of VAMT notably shared 76-83% identity to known plant GABA transaminases originating from *Arabidopsis thaliana*[10], apple [15] and tomato [11]. None of these enzymes have been investigated for the ability to convert vanillin or vanillylamine. In fact, transamination of vanillin has, to the best of our knowledge, only been reported for the ω-transaminase from *Chromobacterium violaceum* DSM 30191 (CV-ωTA) [13]; however, transamination of numerous aryl substituted aldehydes and ketones have previously been described for ω-transaminases originating from various soil-living bacteria, for example ω-TAs from *Aspergillus terreus*, *Hyphomonas neptunium,* and *Arthrobacter* ATA-117 [14,19]. It is likely that there are several other transaminases unrelated to VAMT that are able to convert vanillin to vanillylamine.

### Potential for biocatalytic transaminations

To evaluate whether VAMT has any potential for biocatalytic transamination for the preparation of chiral amines, kinetic resolution of (*rac*)-1-phenylethylamine (1-PEA) was examined and which enantiomeric selectivity the enzyme possesses. The kinetic resolution of (*rac*)-1-PEA using pyruvate as amine acceptor was initiated by addition of the enzyme and was allowed to continue for 24 hours. The products were extracted with heptane and analysed using chiral HPLC. (*S*)-1-PEA was fully converted into acetophenone, while (*R*)-1-PEA remained, leading to an ee > 99% at 50% conversion of (*rac*)-1-PEA. Alike VAMT, most known transaminases that have been used for the generation of chiral amines are *S*-selective [14], with a few exceptions of *R*-selective enzymes [20,21].

**Figure 3:**
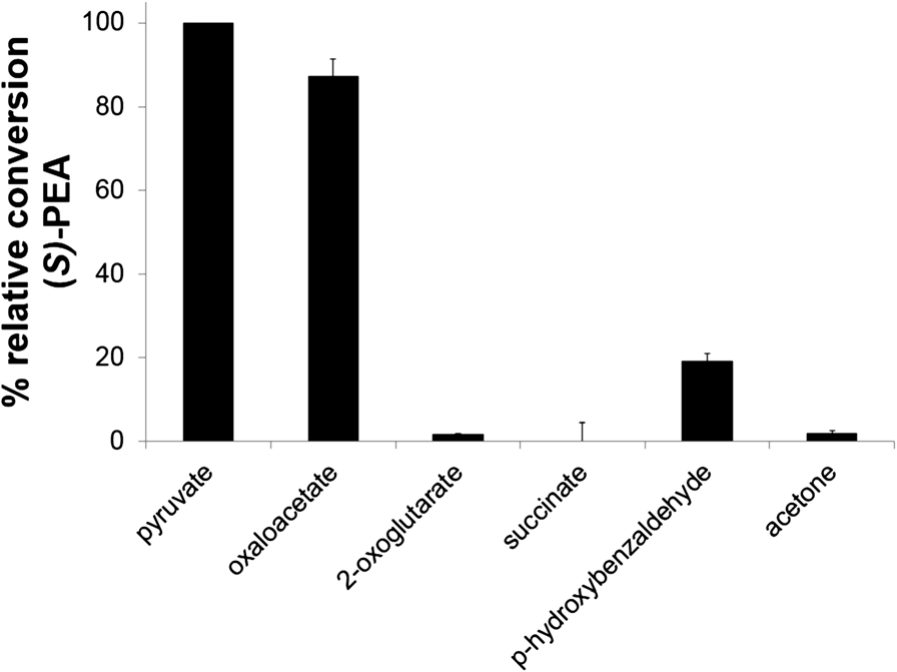
**Relative conversion of (*S*)-1-phenylethylamine ((*S*)-1-PEA) with different amine acceptors.**

To investigate which co-substrates were accepted by VAMT, kinetic resolution with different amine acceptors was carried out (Figure 3). In addition to pyruvate, it was shown that oxaloacetate was also accepted as co-substrate by the enzyme. The enzyme was not able to use 2-oxoglutarate as amine acceptor, which is in accordance with other known GABA transaminases of plant origin [10,11,15]; nor was it able to use succinate or acetone as amine acceptor. However the aromatic aldehyde *p*-hydroxybenzaldehyde displayed some reactivity.

**Figure 4:**
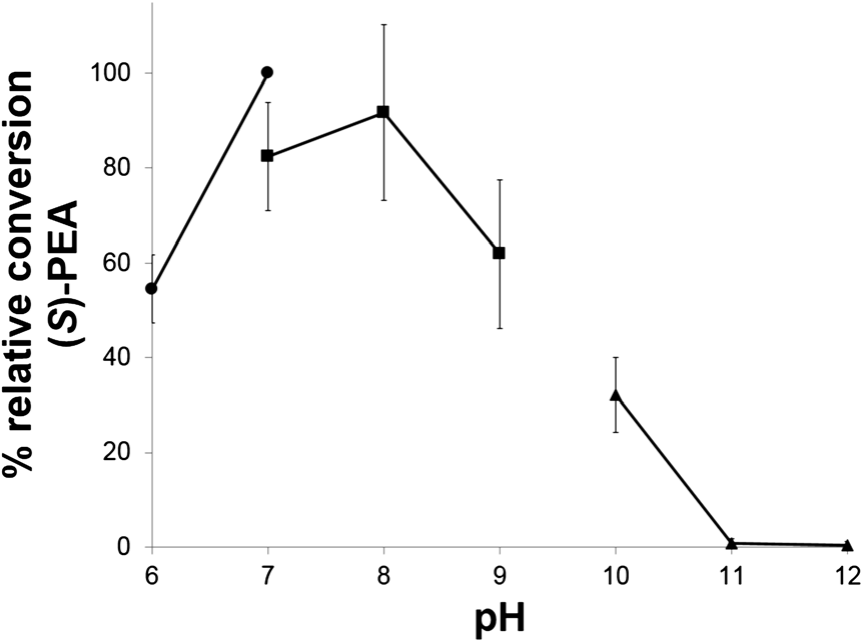
**Relative conversion of (*S*)-1-phenylethylamine at different pH ranging from 6 to 12.** (●) Sodium phosphate buffer; (■) TRIS buffer; (▲) CAPS buffer.

The optimal pH for VAMT activity was investigated by following the relative conversion of (*S*)-1-phenylethylamine to acetophenone at different pH between 6 to 12 (Figure 4). Noteworthy, the enzyme displayed activity over a broad pH range (pH 6-10), and had the highest activity at pH 7-8, which is rather unique in comparison with several other transaminases that have a higher pH optimum [20,22-27]. The high activity at physiological pH demonstrates that VAMT is suitable for whole-cell transamination systems where the reaction takes place in the cytoplasm of viable and metabolically active cells.

## Conclusions

For the first time, the catalytic activity of vanillin transaminase from chili pepper plant *Capsicum chinense* was demonstrated with the purified enzyme *in vitro*. The enzyme displayed high transaminase activity towards vanillylamine and (*S*)-1-phenylethylamine with pyruvate or oxaloacetate as amine acceptor, and the enzyme was active at a broad pH range. Thus, VAMT can be used for the preparation of chiral amines, and represents an interesting candidate to be used in whole-cell biocatalysis with recombinant microorganisms.

## Methods

### Strains and cultivation conditions

*E. coli* strains and plasmids used in the study are listed in Table 1. *E. coli* strain DH5α was used for subcloning and transformants were selected on lysogeny broth (LB) media supplemented with 100 mg/l ampicillin (IBI Shelton Scientific, CT, USA). Strains were kept in 20% v/v glycerol stocks at -80°C and grown for 1 day on LB plates at 37°C before use. A single colony was picked and pre-cultured overnight in LB media and used for subsequent inoculation.

### Strain construction

Standard molecular biology techniques were used for all cloning procedures [28]. Plasmid DNA was prepared with the GeneJET™ Plasmid Miniprep Kit (Fermentas UAB, Vilnius, Lithuania). Agarose gel DNA extraction was made with QIAquick® Gel Extraction Kit (Qiagen GmbH, Hilden, Germany). Sequencing was performed by MWG-Biotech AG (Ebersberg, Germany). Restriction endonucleases and T4 DNA Ligase from Fermentas were used for DNA manipulation. Transformation of *E. coli* was performed with the calcium chloride [29] method.

The VAMT gene from *C. chinense* [GenBank: AAC78480.1, Swiss-Prot: O82521] was constructed synthetically (GenScript, NJ, USA), resulting in plasmid pUC57 VAMT (Table 1). The plasmid was digested with restriction enzymes *Bam*HI and *Pst*I, and the resulting fragment was inserted into pRSETB (Invitrogen, CA, USA). The resulting plasmid pNW1 was digested with *Bam*HI, treated with Klenow fragment from Fermentas and re-ligated with T4 DNA Ligase from Fermentas to move the gene in frame. Correct orientations and sequences of the inserts were verified by restriction analysis and sequencing. pNW1 was transformed into *E. coli* BL21(DE3) pLys which gave strain TMB2100.

### Cell growth

A single microbial colony of TMB2100 from a LB plate [30] containing 100 mg/l ampicillin was inoculated in 50 ml LB medium containing 100 mg/l ampicillin. Cells were grown overnight (at least 16 hr) in an incubator at 37°C with shaking at 180 rpm. The preculture was used to inoculate 250 ml LB medium containing 100 mg/l ampicillin in a 1 l shake flask with an optical density (OD_620_) of 0.1 and growth was performed at 37°C and 180 rpm. Expression of the transaminase gene VAMT was induced by addition of isopropyl-β-D-thiogalactoside (IPTG) to a final concentration of 1 mM when the OD_620_ of the culture reached 0.6 to 0.8 and incubation was then continued for 24 hours at 15°C and 180 rpm.

### Purification of the transaminase

Cells of recombinant *E. coli* strain TMB2100 were harvested by centrifugation (3200 × *g*, 10 min) and washed once with 25 mL water. The cell pellet was resuspended in equilibration buffer, according to the manufacturer’s instructions, and subjected to sonication in a sonicator (Bandelin electronics, Berlin, Germany). Cell debris was removed by centrifugation (3200 × *g*, 10 min). The clear lysate supernatant was collected and passed through a nickel column (HisPur™ Ni-NTA Purification Kit, Thermo Scientific, Rockford, IL, USA), according to the description of the manufacturer. The purified transaminase was collected after elution with buffer, according to the manufacturer’s instructions. Protein concentration was determined using the Bradford method with bovine serum albumin (BSA) as a standard [31]. Purity of the protein in the eluent was estimated with SDS-PAGE (Figure 2). The identity of the protein was confirmed by mass spectrometry at the SWEGENE proteomics platform (Lund, Sweden). The concentration of the purified transaminase was approximately 0.5 mg/ml.

### Enzyme activity assay

Purified VAMT (0.5 ml) was mixed with 4 mM vanillylamine, (*rac*)-1-phenylethylamine (1-PEA) or vanillin, PLP (0.1 mM), and one of the following co-substrates (40 mM for amine acceptors, 100 mM for amine donors): pyruvate, oxaloacetate, 2-oxoglutarate, succinate, *p*-hydroxybenzaldehyde, acetone, L-alanine, D-alanine, GABA, glutamine or ornithine. The reaction was performed in 1 ml sodium phosphate buffer (100 mM, pH 6-7), Tris-HCl buffer (100 mM, pH 7-9) or CAPS buffer (100 mM, pH 10-12) in a glass vial. The reaction was started by the addition of the substrate and it was quenched by the addition of 0.1 ml 1 M HCl. Samples were kept at -20°C until analysis. Thawing of samples (0.5 ml) was done at room temperature and 1-PEA and acetophenone (ACP) were extracted with 0.6 ml heptane, after addition of 30 μl NaOH (10 M), and analyzed by HPLC. The specific activity of the enzyme was calculated based on the rate of vanillylamine conversion (1 U = conversion of 1 μmol vanillylamine per minute). Experiments were done at least in duplicates. The enzyme has a selectivity factor, E > 100 which was calculated according to $E=\frac{\text{ln}\left[\left(1-c\right)*\left(1-\text{ee}\right)\right]}{\text{ln}\left[\left(1-c\right)*\left(1+\text{ee}\right)\right]},$ where c = conversion, ee = enantiomeric excess).

### HPLC analysis

Quantitative analysis of vanillin and vanillylamine were performed with a 1100 Series HPLC instrument (Agilent Technologies, Heilbronn, Germany) equipped with a quaternary pump, autosampler, diode array detector (DAD), fluorescence detector and a vacuum degasser. Chromatographic separation took place in a Zorbax SB-C18 (2.1 mm × 150 mm × 3.5 μm) column (Agilent, USA). A gradient elution method was optimized using two mobile phases, A (0.5% formic acid in water) and B (0.5% formic acid in methanol). The elution gradient started with an initial composition of 4% B and increased to 96% B within 5 min. The late composition was kept isocratic for 7 min, followed by returning back to initial composition in one minute. The method was ended with a 12 min equilibration step at the same composition resulting in a 25 min total runtime. Using the autosampler syringe, 5 μl sample was injected to the column. The mobile phase flow rate kept constant at 0.2 ml/min while monitoring with DAD at 280 nm wavelength. Processing the HPLC data was done using Agilent Chemstation revision B.01.03 software.

(*R*)- and (*S*)-1-phenylethylamine and acetophenone were determined using a Waters HPLC system (Binary HPLC pump 1525, UV/Vis detector 2489, Autosampler 2707) equipped with a Daicel ChiralCel OD-H column (4.6 × 25 mm, 5 μm). The mobile phase was 85:15 heptane/isopropanol with 0.1% butylamine and the flow rate was kept constant at 1 ml/min while monitoring at 210 nm wavelength. Using the autosampler syringe, 5 μl sample was injected to the column. All sample analyses were performed at room temperature. Retention times: (*R*)-1-phenyethylamine: 5.6 min; (*S*)-1-phenylethylamine: 6.8 min; acetophenone: 4.6 min.

## Competing interests

The authors declare that they have no competing interests.

## Authors’ contributions

NW carried out the strain constructions, enzyme purification and characterisation, participated in the design of the study, and drafted the manuscript. AI performed the HPLC method optimisation. MGG participated in the design of the study and its coordination. MC conceived the study, and participated in its design and coordination and helped to draft the manuscript. All authors read and approved the final manuscript.
